# Dr. Kinglake's Reply, on Gout

**Published:** 1803-05-01

**Authors:** Robert Kinglake

**Affiliations:** Taunton


### *To the Editors of the Medical and Physical Journal.*

Gentlemen,

NOT a page of your useful Journal should be misemployed, in giving place to any animadversions from me, on the attack which your "Constant Reader" has made in your last Number (p. 362) on my mode of treating Gout, did it not derive some claim to notice, both from your having admitted it into your publication, and from the discouraging impression it may possibly make on the timid and the prejudiced.

It must be readily perceived, that the communication breathes a spirit of levity and wrangling, wholly inconsistent with the grave decorum due to the investigation and decision of a philosophical subject; nor does the veil of anonymous concealment, which the author has thought fit to assume, impart to the statement the genuine stamp of unquestionable authenticity.

*Hearsay reports* are not legitimate documents for determining the merits of a scientific inquiry. What principle in in either physics or morals would be secure, if assailable by the detracting insinuations of adverse and unsubstantiated rumour?

The names of Harvey and Gregory are adduced to discredit a practice which their superior intelligence led them to adopt in their own cases. Could a stronger proof be given of the firm persuasion they had of its salutary powers? Nor is the supposed shade of the narrative its gloomy part, for it is not pretended that either of these eminent men ever repented what they had done, or in any degree disparaged its beneficial efficacy. If trembling spectators, and commentators, like your "Constant Reader," should imagine dangers where none exist, and assign causes for deaths which not even a *casual co-incidence* of circumstances fully warrants, such groundless dread ought not to repress a humane endeavour to inculcate a rational mode of curing an hitherto untractable malady. It has never been my object to assume credit for either originality, or peculiarity, in reducing the morbid excess of temperature in arthritic affection by diminished heat. It is impossible the principle should have escaped the earliest reasoning on the subject. The principle of the practice may therefore be rather considered as *common* to human intelligence than *peculiar* to any individual. The doctrine of distempered heat at once pervades, and constitutes the most intelligent and instructive parts of Hippocrates's writings. The medical principles and practice of Sydenham also, founded on temperature, formed a transcendant epoch in the history of curative medicine; and happily for mankind, finally overthrew the fatal delusions of humoral pathology, and alexipharmic jargon. Conducted then by analogy, it occurred to me as highly reasonable, that Gout, distinguished like other inflammations by excessive heat, and marked by no essential difference, might be subdued in a similar manner. This persuasion induced me to assimilate the treatment. Not a single fact had previously reached my knowledge to authorize the trial; though, undoubtedly, many were extant. To me therefore the practice was relatively, though not absolutely original. The only claim to orignality which seems to exist in my right, and the only one which deserves a moment's solicitude to establish, is that of publicly recommending the practice, after having experienced its salutary effects in numerous instances, in which the treatment was conducted with such disguise and secrecy, as were necessary to obviate the prohibitive influence of prevailing prejudices against it.

In my estimation, nothing can be more degradingly futile, or ridiculously absurd, than captiously cavilling at a fair attempt to be useful, or to deny merit to those who, at all risks, aim at a practical improvement by patient experiment, and respectful publication of the result.

Your Correspondent, Mr. Wadd,* has my implicit credit in averring his long familiarity with the topical use of cold water in the cure of Gout; but how is his zeal for the improvement of the healing art to be appreciated, if confident of the salutary powers of reduced temperature in that disorder, he did not boldly adopt it in his private practice, and recommend it to public attention?

He says, he could never prevail on any patient to submit to the treatment. It was not likely that he should. What patient will accede to a proposed remedy, that has the reputation of being *certainly* pernicious?

The efficacy of the treatment might have been subjected to the test of experiment, without alarming existing prejudices. Indeed, it should be an invariable rule, in investigating the effects of a new remedy, cautiously to conceal the nature and object of the trial, to prevent its specific operation being disguised by the influence of imagination.

Since my last communication on the cure of Arthritis, the previous stock of my experience has received considerable addition from correspondents, bearing the strongest testimony to the speedy, safe, and effectual remedy, afforded to Gout, by the application of cold water. One case has also since occurred in my own practice, more violently, extensively, and critically circumstanced, than any former instance in my observation; in which, the incessant employ of cold water, partly by bathing, and partly by keeping the affected joints enveloped with cloths wetted in that fluid, completely extinguished the arthritic temperature, and restored the inflamed parts to perfect ease, and tolerable motion, in the course of three days. Friction has since fully renovated the motive power.

Firmly convinced of the inestimable benefit that will accrue to mankind from a *liberal* investigation of the curative power of topical cold to arthritic inflammation, the Medical Faculty will excuse my again soliciting the earliest intelligence of their experience on the subject, that an opportunity may soon be afforded me of presenting the public with such information, as will unequivocally prove Gout,

---

* See Medical and Physical Journal, No. XLIX. p. 205.

Gout, like all other descriptions of inflammation, to be a disease of *excessive temperature*, and agreeably to an evident law of nature, most appropriately, promptly, and efficaciously remediable, by *diminished heat*. Time will elucidate and confirm this opinion.

Magna est veritas, et prævalebit.

*Taunton*, *April* 9, 1803. I am, &c. ROBERT KINGLAKE.

---

